# Production of Sensorily Acceptable Pasta Filata Cheese with Partial Substitution of Sheep’s Milk Powder in Different Forms

**DOI:** 10.3390/foods12091766

**Published:** 2023-04-24

**Authors:** Jakub Biegalski, Dorota Cais-Sokolińska

**Affiliations:** Department of Dairy and Process Engineering, Faculty of Food Science and Nutrition, Poznań University of Life Sciences, ul. Wojska Polskiego 31/33, 60-624 Poznań, Poland; jakub.biegalski@up.poznan.pl

**Keywords:** sheep’s milk powder, consumer acceptability, mozzarella cheese, water-fat serum

## Abstract

The presented study analyzed the possibility of pasta filata cheese production using sheep’s milk powder in different forms and substitution amounts with fresh cow’s milk. For the production of the pasta filata cheeses that were analyzed in the research, sheep’s milk powder and reconstituted sheep’s milk were used for partial substitution with fresh cow’s milk in the amount of approx. 20, 30 and 40 percent (*v*/*v*). The obtained results showed that the more sheep’s milk in the form of powder in the mixture, the lower the cheese’s moisture content. The fat and protein content in the whey after the production of cheeses from mixtures was lower than after the production of cheeses from reconstituted sheep’s milk only. Cheeses produced entirely from reconstituted sheep’s milk displayed the highest fat loss. The greatest cheese yield was observed for cheeses from mixtures with sheep’s milk powder and entirely from reconstituted sheep’s milk. Pasta filata cheeses made from a mixture of cow’s milk and sheep’s milk powder that was not reconstituted were much less acceptable to consumers than reconstituted milk powder cheeses, especially those with 40% and 30% added powder. Sensory profile analysis showed that the addition of sheep’s milk to the mixture, regardless of the form, affected the appearance, consistency, and flavor of the produced pasta filata cheeses. Mixing cow’s milk with sheep’s milk powder created the possibility of modeling the final cheese quality and yield.

## 1. Introduction

Pasta filata cheeses are made from cow, buffalo, goat, and sheep’s milk, and typically come from Eastern Europe, Turkey, Greece, the Balkans, and Italy. The term “pasta filata” originates from the Italian language and means “stretched curd”. Some of the cheeses associated with this group are soft or semi-soft cheeses [1]. Pasta filata cheeses made from cow’s milk are, for example, Fior di Latte and Provolone del Monaco cheese [2,3]. Sheep’s milk is used to produce, among others, Oscypek PDO cheese and Kashkaval cheese [4,5]. Some of these cheeses can be produced using mixtures of milk from different mammals. For example cow’s milk can be mixed with buffalo’s milk to produce mozzarella cheese. It should be remembered that the use of specific types of milk for some cheeses is often dictated by the relevant laws, especially when cheese has Protected Designation of Origin (PDO) or other indications dictated by law. Pasta filata cheeses can be consumed as fresh cheeses or after a short maturation period. For all pasta filata cheeses, the stage of structuring the casein fibers is unique. During this stage, the curd is dipped into hot water or hot, salted brine, followed by kneading (stretching) by hand or mechanically, to obtain a semi-liquid, elastic consistency, which can be molded in various ways [1].

Pasta filata cheese production is often artisanal, although many are industrially produced. To ensure the continuity of production, constant supplies of raw materials are necessary, and in the case of sheep’s milk this is difficult due to the seasonal nature of its production [6]. Freezing sheep’s milk could resolve this problem, but research has shown that the use of frozen/thawed sheep’s milk for pasta filata cheese production was not conducive to positive effects related to technological, economical, and sensory aspects [7]. Cheeses produced using frozen/thawed sheep’s milk are not fully accepted by consumers. The addition of frozen/thawed milk resulted in reduced stretching, which is one of the main and most characteristic features of pasta filata cheeses [7]. Therefore, cheese production technologies based on raw materials that were previously collected and preserved are constantly being sought. One of the methods of preserving milk is the drying method.

Milk powder is very often used in food production, mainly because of its functional properties and nutritional value [8,9]. Additionally, it is an important ingredient in many ready-to-eat (RTE) products, such as granola, infant milk formula, protein bars, and seasonings [10]. Dairy proteins from milk powder exhibit gelation, emulsification, and foam-formation properties [11]. Furthermore, the conversion of milk solids into powder extends their shelf-life at ambient temperatures, which is mainly due to the applied temperature treatment and reduced water activity [11]. This extended time allows for the rational use of milk powder in accordance with the current demand. In addition, reconstitution and/or recombination of the milk allows for the adjustment/combination of the dry matter content and/or the protein content of the milk after the addition of water. Reconstituted milk in liquid form results from combining skimmed or whole milk powder with water in an amount that provides the right ratio of water to solids [12]. Additionally, this milk is a very important material in dairy production, especially in regions of the world where access to fresh raw milk is limited [12]. Reconstituted milk produced from milk powder can be used to partially or completely replace fresh milk in cheese production [13].

To the best of our knowledge, to date, there is no information regarding the possible use of sheep’s milk powder in the production of pasta filata cheese. Herein, we hypothesized that it is possible to produce sensorily acceptable fresh pasta filata cheese using sheep’s milk powder in different forms and substitution amounts with cow’s milk. For this purpose, we conducted technological tests and instrumental analysis. The obtained results were combined with descriptive sensory analysis of the produced cheeses. The performed experiments determined the proper amount of substitution of sheep’s milk (reconstituted/in form of powder) in a mixture with fresh raw cow’s milk. In addition to cognitive purposes, we developed an efficient production protocol that allowed the estimation of cheese yield using mathematical formulas that are well known and traditionally used in the dairy industry. Understanding the phenomena, in conjunction with the technological data, can affect the transfer of scientific effects to real production in a dairy plant.

## 2. Materials and Methods

### 2.1. Milk Samples

The research material used in this study was fresh raw cow’s milk (C), sheep’s milk powder (SP), reconstituted sheep’s milk from milk powder (SR), and their mixtures in different configurations (CSP and CSR).

Highly hygienic and cytological quality Holstein-Friesian cow’s milk intended for dairy plants was used. Sheep’s milk powder was purchased as a commercial product (Les Jardins de sainte Hildegarde, Coux et Bigaroque, France) and had the following parameters (g/kg): dry matter 962.0, water 38.0, non-fat solids 604.0, fat 358.1, total protein 309.9, casein 252.6, whey protein 57.3, lactose 258.2, and ash 33.5. The bulk density of the sheep’s milk powder was 0.344 g/cm^3^. Milk powder was used for the production of reconstituted sheep’s milk by mixing with hot water (145 g/L, 60 °C, 90 s, 36 rpm) in a double-coat cheese kettle, type SKM50, equipped with automatic propeller stirrer (Plevnik d.o.o., Dobrova, Slovenia).

Milk powder and reconstituted milk were used for partial substitution with fresh raw cow’s milk in the amount of approx. 20, 30, and 40 percent (*v*/*v*). The exact amount of sheep’s milk powder and reconstituted sheep’s milk added to the fresh raw cow’s milk (*w*/*v*) is presented in Table 1. The listed amounts allowed us to obtain reconstituted milk with a composition comparable to raw cow’s milk. The sample coding presented in Table 1 was used throughout the manuscript.

### 2.2. Cheese-Making Protocols

A detailed description of fresh raw cow’s milk used in the production process and the procedure of making pasta filata cheese was described by Biegalski et al. [7]. The entire experiment was repeated 6 times (*n* = 6). Within each production batch, 7 cheeses were produced. Each time, 70 L of milk was used in each batch. The produced cheeses were shaped into spheres (220 g, Ø = 7 cm). The cheese was packed in brine and stored at 3 °C. PA/PE bags with a thickness of 0.08 mm were used. The various parameters of cheese quality and sensory tests were rated after 2 days from the production date, which imitated the period of time from the end of production to the moment the product goes on sale (storage in packaging at 3 ± 0.5 °C). The samples were taken from different production batches (*n* = 6). The cheese was prepared in a pilot plant scale, and each batch was analyzed twice.

### 2.3. Pasta Filata Cheese Yield Calculation

Pasta filata cheese yield was calculated using Equations (1)–(6) [14,15] using mean values. Pasta filata cheese yield was also interpreted using Equation (7), created on the basis of the *MY* variable described by Sales et al. [16]:

Van Slyke and Price Equation (*X_SP_*):

(1) $$X_{SP}=1.63\;\left(f+c\right),$$

McDowall Equation (*X_MD_*)

(2) $$X_{MD}=1.07f+2.35c,$$

Herz Equation (*X_H_*):

(3) $$X_{H}=\frac{100\left(f+3\right)}{100-w},$$

Rinckleben Equation (*X_R_*):

(4) $$X_{R}=\frac{100\left(f-f^{\prime }+0.30r\right)}{100-w},$$

Jakubowski Equation (*X_J_*):

(5) $$X_{J}=\frac{100\left(f-0.9f^{\prime }+1.03\times c\right)}{100-w},$$

Pijanowski Equation (*X_P_*):

(6) $$X_{P}=\frac{f-f^{\prime }+c}{1-\frac{w}{100-r^{\prime }-f^{\prime }}},$$

*MY* variable:

(7) $$MY=\frac{m_{u}}{ch_{p}},$$

where: *f* = fat content in milk (%); *f*’ = fat content in whey (%); *r* = non-fat solids content in milk (%); *r*’ = non-fat solids content in whey (%); *c* = casein content in milk (%); *w* = water content in cheese (%); *m_u_* = amount of used milk (kg); *ch_p_* = amount of produced cheese (kg).

### 2.4. Composition

A Bentley DairySpec FT Manual (Bentley Instruments, Inc., Chaska, MN, USA) was used to determine the composition of the studied milk. The composition of the cheese was determined according to moisture [17], protein [18], and fat [19] content. Total protein was calculated as: (TN − NPN) × 6.38, where TN is total nitrogen and NPN is non-protein nitrogen.

### 2.5. Acidity

pH was measured using a CP–402 pH-meter (Elmetron, Zabrze, Poland) equipped with a IONODE IJ44A electrode (Ionode Pty. Ltd., Tennyson, Australia). The titratable acidity values were expressed as Soxhlet–Henkel degrees (°SH).

### 2.6. Sensory Analysis

For sensory analysis, the profiling method [20,21] was used. The panel comprised a team of 13 individuals (aged from 22 to 55), prepared to perform sensory examinations [22,23]. Before examinations, each panel member was adequately trained for 36 h in total. Samples were evaluated using 8 cm unstructured line scales anchored with terms “low” (as undetectable) at the left, and “high” (as very intense) at the right. To describe the flavor of the cheese, the terms presented in Table 2 were used during the descriptive analysis.

In the evaluation of the overall desirability of the produced cheese, consumers (*n* = 102; 53 female, 49 male; age from 22 to 67; M_age_ = 34.0, SD = 9.57) were asked to indicate how much they liked or disliked each product on a 9-point hedonic scale (9 = like extremely; 1 = dislike extremely). Each consumer was given 7 cheese samples for evaluation. Each cheese sample was made from reconstituted sheep’s milk or a mixture of sheep’s milk powder/reconstituted sheep’s milk with fresh raw cow’s milk, as shown in Table 1. The samples were assessed 24 h after production. Samples were held and served at 6 °C in a refrigerated display case (YG-05025, YATO, Wrocław, Poland).

Each consumer (*n* = 102) who took part in the overall desirability evaluation was also asked to rate appearance (whey leachate), consistency (springiness), aroma (creamy), and flavor (salty) using a 5-point just-about-right (JAR) scale. For this purpose, the methodology described by Costa et al. [24] was used. Ratings consisted of 1 = not enough, 3 = ideal, 5 = too much.

### 2.7. Statistical Analyses

Standard error of the mean (SEM) was used for the presentation of results. Some results are also presented as the mean ± standard deviation (SD) in triplicate of each analysis carried out in experiments performed in duplicate. A critical level of significance of α = 0.05 was used throughout the study. The influence of the partial substitution of sheep’s milk powder/reconstituted sheep’s milk on the characteristics of pasta filata cheeses was evaluated by one-way analysis of variance (ANOVA). The results of the determination of the position of the tested samples in the perception of the space were evaluated using principal component analysis (PCA) based on the NIPALS algorithm. Sensory profile results were presented using principal component analysis (PCA) to check the correlation between the parameters. Statistical analysis was carried out using TIBCO Statistica data analysis software, version 13.3.0 (TIBCO Software Inc., Palo Alto, CA, USA).

## 3. Results and Discussion

### 3.1. Composition and Physicochemical Properties of Milk and Fresh Pasta Filata Cheese

Significant differences in composition and technological parameters were observed in the analyzed milk mixtures (Table 3). The mixtures of cow’s milk and unreconstituted sheep’s milk (CSP) compared to the mixtures of cow’s milk and reconstituted sheep’s milk (CSR) were characterized by a significantly higher content of all tested components. The greater the addition of sheep’s milk powder in both CSP and CSR mixtures, the higher the content of components. Hence, the highest content of individual components was found in sample CSP6/4. CSP mixtures contained more non-fat solids compared to SR milk. For example, the non-fat solids content in sample CSP6/4 was 135.1 g/kg, which was almost 60% higher than that reported for CSR6/4 sample and approx. 35% higher than the value for reconstituted sheep’s milk SR (*p* < 0.05). The same result was obtained for total protein, which was also the highest for the CSP6/4 mixture. The casein content in the total protein of CSP and CSR mixtures was 80.8% and 80.7%, respectively. In the CSR mixtures, it was shown that the higher the share of reconstituted sheep’s milk, the lower the lactose content. However, this was in contrast to total protein and non-fat solids content. The lactose content in CSR mixtures ranged from 40.4 to 42.5 g/kg (Table 3). Interestingly, the ratio of fat to total protein (1.3) in the mixtures of cow’s milk with sheep’s milk powder in the proportions of 6/4 and 7/3 was similar and independent of whether milk powder or reconstituted milk was used. The less sheep’s milk powder (regardless of the form) present in the mixture, the higher the ratio of fat to total protein (1.5). This was due to the higher proportion of cow’s milk in the mixture. Tavakoli et al. [25] showed that reconstituted cow’s milk had a total protein content of 3.4% to 3.5% (approx. 34–35 g/kg), which was more than 50% lower than for the sheep’s milk analyzed in our work. Tavakoli et al. [25] showed also that reconstituted cow’s milk had lower fat content (approx. 66%) and very similar pH and non-fat solids content (for full-fat reconstituted milk) (Table 3). The analysis of the results indicated that pH and titratable acidity were typical for milk, although SR milk had a titratable acidity significantly higher compared to the other samples. The composition and technological parameters of fresh raw cow and sheep’s milk have been previously presented [7]. Values of composition and technological parameters that were comparable to raw cow’s milk were observed for CSR mixtures, especially for CSR7/3 and CSR8/2 mixtures. This proved that sheep’s milk is characterized by a higher content of total solids and major nutrients compared to cow’s milk, as described by Chia et al. [26].

**Figure 1:**
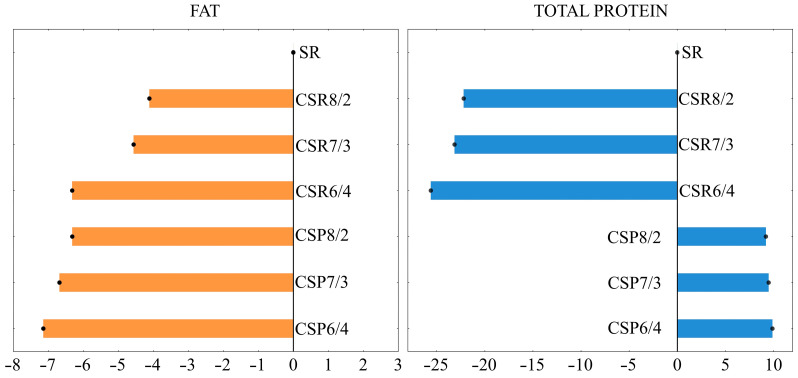
Deviation of fat and total protein content from the value shown for pasta filata cheese from SR milk (%). Coding as in Table 1.

The basic composition and physicochemical properties of milk mixtures impacted the characteristics of the produced pasta filata cheeses (Table 4). The moisture content in cheeses made of milk mixtures was higher than in those made of SR milk (*p* < 0.05). Additionally, the greater the share of sheep’s milk in the mixture, the lower the moisture content of the cheese, but only when sheep’s milk was added in the form of powder (CSP6/4; 507.6 g/kg). When sheep’s milk was added to the mixture in reconstituted form, a higher moisture content was observed. Cheeses made from CSP mixtures had moisture content in the range of 507.6 to 511.1 g/kg. However, those made with CSR mixtures had a moisture content ranging from 583.8 to 594.8 g/kg. Moghiseh et al. [27] showed that the moisture content of fresh cow’s milk mozzarella cheese was 52.2%. This value was almost 14% lower than that presented for cheeses made of CSR mixtures in our work. The closest moisture content to that of cow’s cheese is the moisture of cheese from the CSR8/2 mixture. It is also worth noting that this value was higher than that reported for raw sheep’s milk cheeses [7]. The fat content in the cheeses from CSP and CSR6/4 mixtures had an average value of 181.4 g/kg, which was lower than in the cheeses made from other CSR mixtures, and even more than SR milk cheese (*p* < 0.05; Figure 1). Despite the protein content in the mixtures used for cheese production being higher after the addition of SR milk (Figure 1), the final ratio of protein to fat in cheeses from the CSR mixture was similar. This was also observed for CSP mixtures, although the protein-to-fat ratio increased from 1.0 (CSR) to 1.5 (CSP). Tidona et al. [28] showed the content of fat (17.1 g/100 g) and protein (16.5 g/100 g) for cow’s mozzarella cheese from a mixture of 40 g/100 g reconstituted and fresh milk. These values were lower than those we found for sheep’s milk, but comparable to cheeses from CSR mixtures, which contained fat in the range of 182.0 to 186.3 g/kg, and protein in the range of 184.3 to 192.77 g/kg.

**Figure 2:**
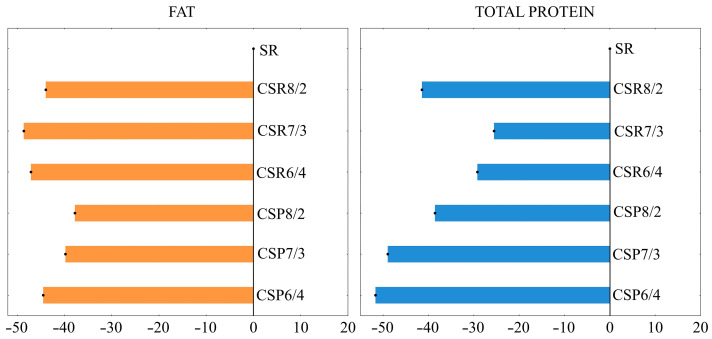
Deviation of fat and total protein content from the value shown for whey remaining after the production of pasta filata cheese from SR milk (%). Coding as in Table 1.

The composition and physicochemical properties of whey remaining after the production of pasta filata cheeses are presented in Table 5. After cheese production, the largest amount of non-fat solids was found in whey from CSR8/2 mixture cheeses (51.6 g/kg), which was approx. 1.6% higher than that from SR milk cheeses. The content of fat and protein in whey from CSP and CSR mixtures cheeses was significantly lower than that in whey from cheeses produced from SR milk (Figure 2). A contrasting relationship was observed for lactose content, where its share in non-fat solids in whey from all mixture cheeses was, on average, 80.2% and was higher than in SR milk whey (74.7%). The highest fat loss in whey was detected for cheeses produced from SR milk. For cheeses produced from CSP and CSR mixtures, fat loss in whey was 2.9% and 3.8%, respectively, in comparison to milk fat. The highest fat loss in whey occurred in the case of cheese from CSR mixtures, and the lowest fat loss was for cheese from the CSP6/4 mixture (2.4%). Denaturation changes in the protein membranes surrounding the fat globule should also be considered, and the number of fat globules of different diameters. Johnson et al. [29] indicated that loss of fat to the whey may be related to the rigidity and structure of the network, which is directly related to the pore and casein aggregate size. The same research group reported that a more porous curd, which was created as a result of a more rigid coagulum, will show a greater release (of fat, among other things) during the pressing of the curd. Tidona et al. [28] showed that whey after the production of cheese from a mixture of fresh and reconstituted milk contained less fat than cheese made from only raw milk. Additionally, they indicated that whey after cheese production from a mixture of fresh and reconstituted milk contained less fat after cheese production than after making cheese from raw milk only. Additionally, Truong et al. [30] indicate that the retention of fat in whey is related to the size of the fat globules.

### 3.2. Suitability of Milk Used for Pasta Filata Cheese Production Based on the Calculation of Yield

Cheeses produced from CSP and SR mixtures were characterized by a higher yield compared to those produced from CSR mixtures (Table 6). The highest yield was determined for cheese from the CSP6/4 mixture. Depending on the equation used (1–6), the yield was 21% (Equation (3)) or even 36%, higher than the yield of cheese from SR milk (Equation (4)). The yield was greater when the addition of sheep’s milk powder in CSP mixture was also greater. The less sheep’s milk was added to the CSR mixtures, the smaller the cheese yield was.

In the case of CSP mixtures, it should be remembered that sheep’s milk was added only in the form of milk powder without prior reconstitution. Thus, the milk was richer in non-fat solids, but at the same time, the water content was much smaller. To produce 1 kg of cheese, considerably more CSP mixture was required than CSR, which was clearly shown by the results for variable *MY* (Equation (7)). In the case of CSP6/4 mixture, 5.67 kg of milk was needed (variable *MY*). This was almost 11% more compared to cheese made from SR milk and almost 31% more compared to cheese made from the CSR6/4 mixture. Ur Rehman et al. [31] reported that the addition of milk protein concentrates can increase cheese yield twofold due to the high recovery of milk solids in the cheese.

The use of Equations (1)–(6) more accurately reflects the content and mutual relations between protein, fat, and non-fat solids in the mixtures produced in the experiment, which may affect the assessment of the cheese yield. The relationship between water content in cheese and non-fat solids content in whey further justifies the choice of equations selected for calculating the yield for cheeses made with reconstituted sheep’s milk (SR and CSR). Regardless of the mutual proportions and the share of ingredients in the dry matter, the *MY* variable can be used, which determines the amount of milk by weight (kg) needed to produce 1 kg of mozzarella cheese. Analysis of the cheese yield calculated using variable *MY*, revealed that the smallest amounts of milk to produce 1 kg of mozzarella cheese were actually for CSR mixtures (Table 6). To produce 1 kg of mozzarella cheese from CSR mixtures, 4.33 to 4.81 kg of the mixture was required. Furthermore, this result was less than for the CSP6/4 mixture cheese (5.67 kg/kg). Francolino et al. [32] reported that the yield of mozzarella cheese produced from milk with addition of milk protein concentrate increased due to a higher recovery of total solids and protein. However, Tidona et al. [13] showed that mozzarella cheese production from a mixture of 40/100 g of reconstituted cow’s milk with fresh cow’s milk did not significantly affect the actual cheese yield compared to cheese produced only with fresh cow’s milk. The use of the variable *MY* for CSR samples compared to CSP indicated the validity of the introduction of sheep’s milk in the form of milk powder after reconstitution. Lower milk usage may hold an important economic aspect for the production process and better use of the production material components from a consumer-nutrition point of view. However, the nutritional value is not the only aspect evaluated by the consumer when choosing a product to buy. Consistent with the positive nutritional aspects, there must also be a sensory aspect and general acceptability.

### 3.3. Analysis of Sensory Profile and Overall Pasta Filata Cheese Desirability

**Figure 3:**
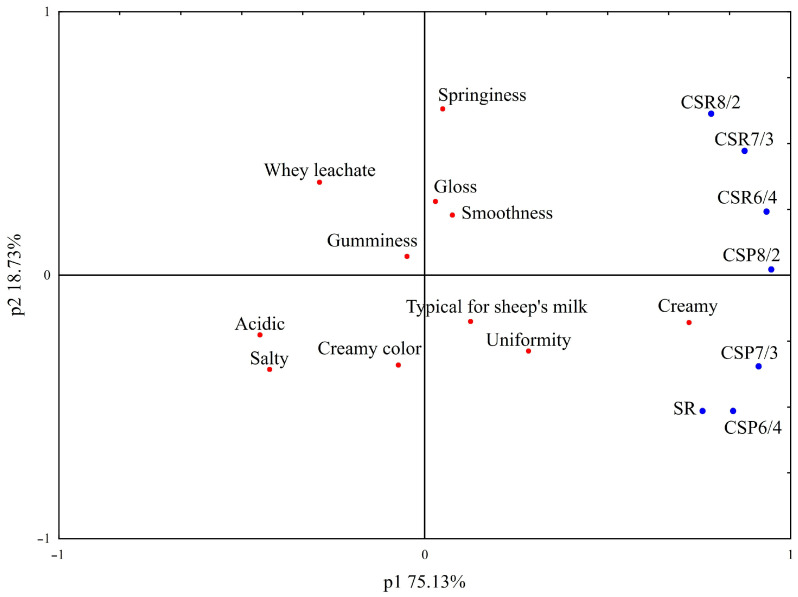
Principal component analysis for the consistency, flavor, appearance, aroma, and mouthfeel of pasta filata cheeses from reconstituted sheep’s milk and mixtures of powder/reconstituted sheep’s milk and cow’s milk in different configurations. Sample coding as in Table 1.

Analysis of the sensory profile showed that pasta filata cheeses from mixtures of sheep’s milk powder in different forms and configurations with cow’s milk differed in terms of appearance, consistency, and flavor (Figure 3). There were no differences in aroma and mouthfeel of the tested samples. Descriptor “creamy color” was rated the higher the more sheep’s milk was added to the mixture used to produce the cheese. This phenomenon was not reliant on the form of sheep’s milk (reconstituted or powder). The opposite relationship was found for the descriptors “smoothness” and “whey leachate”. The less sheep’s milk added to the mixture, the higher these descriptors were rated. Cheese from CSR mixtures was “glossier” (up to 60%) than that from CSP mixtures. No differences were found in the analyzed samples in terms of the consistency descriptor “gumminess”. In contrast, “springiness” was higher for cheeses made from CSR mixtures, even when compared to cheeses made from SR milk. The studied cheeses did not differ in aroma (“creamy” and “typical for sheep’s milk”). However, for flavor, differences were found only in relation to the descriptor “salty”. Furthermore, the saltiness was higher and statistically different only in cheeses made from SR milk and mixtures with 40% sheep’s milk content, regardless of the form used.

Cheeses made from mixtures containing sheep’s milk powder without reconstitution (CSP) were much less accepted by consumers compared to the other samples studied (Table 7). A similar relationship was observed for cheeses that were produced from mixtures with reconstituted milk (CSR), in which the amount of reconstituted sheep’s milk was higher than 30%. Dissatisfaction (dislike) was higher the greater the amount of sheep’s milk powder added to CSP mixture. Dislike responses increased from around 23% for CSP8/2 cheeses to over 98% for CSP6/4 cheeses. In general, almost all consumers disliked CSP6/4 mixture cheeses. In the case of cheeses from CSP7/3 mixture, approx. 84% of consumers gave “dislike” responses.

The main reason for consumer dissatisfaction in the case of pasta filata cheeses produced from CSP mixtures was “not enough” of the following qualities: springiness (consistency) and creamy (flavor), and “too much” of the following qualities: creamy, salty (flavor), and whey leachate (appearance, Table 8). For creamy (flavor), a discrepancy was observed in consumer assessment. For example, for cheese from CSR7/3 mixture, the flavor creamy was “not enough” for almost 29% and “too much” for almost 15% of consumers. The feeling of creamy (flavor) is therefore very individual, but in JAR assessment, most consumers (56%) said that creamy (flavor) was ideal.

Cheeses from CSR mixtures showed a change in springiness (consistency) from “not enough” to “too much”, compared to cheeses produced from CSP mixtures. For none of the cheeses presented to consumers, “not enough” of the whey leachate was noted. “Too much” leachate was most commonly reported for SR milk cheeses, CSR mixture cheeses, and CSP mixture cheeses with 20% sheep’s milk powder content. Importantly, whey leachate, which was noticed by consumers of pasta filata cheese, was lower the greater the amount of sheep’s milk powder added. This was particularly noticeable for cheeses from CSP mixtures.

Sensory analysis showed that the cheeses produced from a mixture of cow’s milk and sheep’s milk powder or reconstituted sheep’s milk differed statistically. Research on the possibility of using the milk of other mammals in a mixture with cow’s milk in the production of pasta filata cheese has previously been reported. For example, Sameen et al. [33] showed that mozzarella cheeses made from a mixture of cow’s milk and fresh buffalo milk were more acceptable than those made only from cow’s milk. Niro et al. [34] presented the possibility of producing caciocavallo cheese from a mixture of fresh cow’s milk and fresh sheep or goat’s milk. These reports proved that cheeses made from a mixture using goat’s milk were, in the opinion of the panelists, more elastic, and cheeses made from a mixture with sheep’s milk had more intense flavor and were saltier compared to cheeses made only from cow’s milk. However, these studies did not examine cheeses from mixtures with sheep’s milk powder or reconstituted sheep’s milk.

## 4. Conclusions

Mixing cow’s milk with sheep’s milk in a reconstituted form in the amount of 20% to 40% or added in the form of powder creates the possibility of modelling parameters of cheese and its yield. The cheeses with the addition of reconstituted sheep’s milk contained less protein and more moisture than those with its addition in the form of powder. The moisture content of these cheeses was even greater than for cheeses made entirely from reconstituted sheep’s milk. It was found that the reconstitution of sheep’s milk powder significantly reduced the yield of cheeses made from mixtures according to the calculations that considered the composition of the mixture. The opposite result was obtained when calculating the volume of the mixture used to produce 1 kg of cheese, so it is more suitable for use in case of pasta filata cheese produced with the addition of milk powder.

The addition of sheep’s milk significantly affected the “creamy color” descriptor of the cheeses, which was greater the more sheep’s milk was added to the mixture (regardless of the form). Additionally, “whey leachate” was greater in the cheese, with less sheep’s milk in the mixture. However, the production of pasta filata cheeses from a mixture of cow’s milk and non-reconstituted sheep’s milk powder was not fully accepted by consumers. The addition of 20% to 40% reconstituted sheep’s milk to cow’s milk during the production of pasta filata cheeses contributed to the improvement of their acceptability. Pasta filata cheeses made from a mixture of cow’s milk and reconstituted sheep’s milk in a ratio of 7:3 were the most liked by consumers. This was despite consumers stating that these cheeses had “too much” springiness, were “too” salty, and had “too much” whey leachate.

Results obtained from the calculation of pasta filata cheese yield can be useful in creating cheeses using sheep’s milk, despite its seasonality of production. Results show the possible increase in cheese yield by use of sheep’s milk powder in both forms: native (powder) and reconstituted. This may be of benefit not only for the producer but also for the consumer. Sensory analysis showed that production of pasta filata cheese using sheep’s milk powder is feasible not only in terms of overall acceptability but also in terms of specific sensory attributes. Therefore, the results obtained highlight the direction of further improvement of the flavor and aroma profile, consistency, and especially the leachate of the water–fat serum of these cheeses.

## Figures and Tables

**Table 1:** Amount of milk powder and reconstituted milk added to the raw milk (*w/v*) during sample preparation.

Sample Codes	Fresh Raw Cow’s Milk (L)	Sheep’s Milk Powder (g/L)	Reconstituted Sheep’s Milk (L)
SR	0.00	0.00	100.00
CSP6/4	60.00	110.50	0.00
CSP7/3	70.00	82.83	0.00
CSP8/2	80.00	55.17	0.00
CSR6/4	60.00	0.00	40.00
CSR7/3	70.00	0.00	30.00
CSR8/2	80.00	0.00	20.00

**Table 2:** Descriptors of cheese subjected to sensory examination.

Attribute Type and Attributes	Definition	Standard/Reference
Appearance		
Creamy color	The property of color as perceived by the eye	1—White color 9—Vanilla custard color
Gloss	The glossiness of a surface observed with reflected light	1—No gloss 9—Water-surface-like gloss
Smoothness	Characteristic related to the uniformity of the surface of the cheese	1—Rugged 9—Smooth
Whey leachate	Presence of water-fat complex leachate coming out of the cheese mass.	1—No leachate 9—Large quantity of leachate (more than one tablespoon)
Consistency		
Gumminess	Attribute characteristic for gelatin-based candy	1—Ricotta cheese consistency ^a^ 9—Gouda cheese consistency ^b^
Springiness	Attribute related to the cheese mass returning to its original state after pressing	1—Gouda cheese springiness ^b^ 9—Gummy bear candy springiness ^c^
Aroma		
Creamy	Aroma typical for cow’s milk cream	1—Cow’s milk cream, 12% fat ^d^ 9—Cow’s milk cream, 36% fat ^e^
Typical for sheep’s milk	Aroma typical for sheep’s milk and its products	1—Cow’s pasteurized milk, 3.5% fat ^f^ 9—Sheep’s pasteurized milk, 5.0% fat ^g^
Flavor		
Salty	Flavor typical for water solution of sodium chloride	1—Mozzarella di bufala campana PDO cheese ^h^ 9—Sheep’s feta cheese ^i^
Acidic	Flavor typical for water solution of citric acid	1—Cow’s milk sweet cream, 36% fat ^e^ 9—Cow’s milk fresh, quark cheese, 4.0% fat ^j^
Mouthfeel		
Uniformity	Perceived uniformity of the sample evaluated in the mouth associated with homogeneity of cheese mass	1—Sheep’s milk powder, 36% fat ^k^ 9—Mozzarella di bufala campana PDO cheese ^h^

^a^ Ricotta cheese (Lactalis Polska Sp. z o.o., Warsaw, Poland); ^b^ Gouda cheese (Spółdzielnia Mleczarska “MLEKPOL”, Grajewo, Poland); ^c^ Gummy bear candy (Haribo GmbH and Co. KG, Grafschaft, Germany); ^d^ Cow’s milk cream, 12% (Spółdzielnia Mleczarska “MLEKPOL”, Grajewo, Poland); ^e^ Cow’s milk cream, 36% (Okręgowa Spółdzielnia Mleczarska w Piątnicy, Piątnica, Poland); ^f^ Cow’s pasteurized milk, 3.8% (Spółdzielnia Mleczarska “MLEKPOL”, Grajewo, Poland); ^g^ Sheep’s pasteurized milk, 5.0% (LEONTEUS, s.r.o., Bratislava, Slovakia); ^h^ Mozzarella di bufala campana PDO cheese (Nuova Castelli S.p.a., Reggio Emilia, Italy); ^i^ Sheep’s feta cheese (Koliós S.A, gr. H ΚOΛΙOΣ A.Ε. Polykastro, Limnotopos Kilkis, Greece); ^j^ Cow’s milk fresh, quark cheese, 4.0% fat (Mlekovita, Wysokie Mazowieckie, Poland); ^k^ Sheep’s milk powder, 36% (Les Jardins de sainte Hildegarde, Coux et Bigaroque, France).

**Table 3:** Composition and technological parameters of reconstituted sheep’s milk and mixtures of powder/reconstituted sheep’s milk with cow’s milk in different configurations.

Material for Pasta Filata Cheese Production								
Parameters	SR	CSP6/4	CSP7/3	CSP8/2	CSR6/4	CSR7/3	CSR8/2	SEM
Non-fat solids (g/kg)	100.0 ^d^	135.1 ^g^	117.7 ^f^	104.3 ^e^	84.7 ^c^	84.1 ^b^	83.6 ^a^	0.006
Fat (g/kg)	59.8 ^e^	77.6 ^g^	67.4 ^f^	58.6 ^d^	48.7 ^c^	47.5 ^b^	47.2 ^a^	0.004
Total protein (g/kg)	51.5 ^e^	61.4 ^f^	51.6 ^e^	38.4 ^c^	38.6 ^d^	37.4 ^b^	30.5 ^a^	0.005
Casein (g/kg)	41.7 ^e^	49.4 ^g^	42.0 ^f^	30.9 ^c^	31.4 ^d^	30.1 ^b^	24.5 ^a^	0.002
Whey protein (g/kg)	9.9 ^d^	11.3 ^e^	9.9 ^d^	7.7 ^c^	7.5 ^b^	7.4 ^b^	5.5 ^a^	0.002
Lactose (g/kg)	42.9 ^d^	65.4 ^g^	57.6 ^f^	52.4 ^e^	40.4 ^a^	41.1 ^b^	42.5 ^c^	0.002
Non-fat solids/Total protein	1.9	2.2	2.3	2.7	2.2	2.2	2.7	
Fat/Total protein	1.2	1.3	1.3	1.5	1.3	1.3	1.5	
Ash (g/kg)	5.4 ^a^	9.3 ^f^	8.9 ^e^	7.7 ^d^	5.5 ^b^	6.5 ^c^	6.5 ^c^	0.003
pH	6.65 ^a^	6.63 ^a^	6.64 ^a^	6.64 ^a^	6.63 ^a^	6.63 ^a^	6.64 ^a^	0.001
Titratable acidity (% lactic acid)	0.214 ^c^	0.186 ^b^	0.176 ^a^	0.172 ^a^	0.184 ^b^	0.176 ^a^	0.171 ^a^	0.000

^a–g^ Means within a row with different superscripts differ (*p* < 0.05). SEM: standard error of the mean (*n* = 6). Sample coding as in Table 1.

**Table 4:** Composition and physicochemical properties of pasta filata cheeses from reconstituted sheep’s milk and mixtures of powder/reconstituted sheep’s milk with cow’s milk in different configurations.

Pasta Filata Cheese								
Parameters	SR	CSP6/4	CSP7/3	CSP8/2	CSR6/4	CSR7/3	CSR8/2	SEM
Moisture (g/kg)	502.8 ^a^	507.6 ^b^	509.2 ^b,c^	511.1 ^c^	594.8 ^f^	587.0 ^e^	583.8 ^d^	1.646
Fat (g/kg)	194.3 ^c^	180.4 ^a^	181.3 ^a^	182.0 ^a^	182.0 ^a^	185.4 ^b^	186.3 ^b^	1.090
Fat/dry matter (*w*/*w*)	0.39	0.37	0.37	0.37	0.45	0.45	0.45	
Protein (g/kg)	247.8 ^d^	272.3 ^e^	271.3 ^e^	270.6 ^e^	184.3 ^a^	190.4 ^b^	192.8 ^c^	1.060
Protein/dry matter (*w*/*w*)	0.50	0.55	0.55	0.55	0.45	0.46	0.46	
Protein/fat (*w*/*w*)	1.3	1.5	1.5	1.5	1.0	1.0	1.0	
Salt (g/kg)	0.45 ^a^	0.46 ^a^	0.46 ^a^	0.47 ^a^	0.47 ^a^	0.47 ^a^	0.47 ^a^	0.000
pH	5.11 ^a^	5.12 ^a^	5.11 ^a^	5.13 ^a^	5.13 ^a^	5.12 ^a^	5.12 ^a^	0.001
Titratable acidity (% lactic acid)	0.711 ^c^	0.709 ^a,b,c^	0.708 ^a,b,c^	0.705 ^a^	0.710 ^b,c^	0.706 ^a,b^	0.705 ^a^	0.000
Water activity	0.9602 ^a^	0.9776 ^e^	0.9771 ^e^	0.9698 ^d^	0.9607 ^a,b^	0.9612 ^b^	0.9621 ^c^	0.000

^a–f^ Means within a row with different superscripts differ (*p* < 0.05). SEM: standard error of the mean (*n* = 6). Sample coding as in Table 1.

**Table 5:** Composition and physicochemical properties of whey remaining after production of pasta filata cheeses from reconstituted sheep’s milk and mixtures of powder/reconstituted sheep’s milk with cow’s milk in different configurations.

Whey Remaining after Cheese Production								
Parameters	SR	CSP6/4	CSP7/3	CSP8/2	CSR6/4	CSR7/3	CSR8/2	SEM
Non-fat solids (g/kg)	50.8 ^c^	49.2 ^a^	49.5 ^b^	51.3 ^d,e^	51.1 ^d^	51.3 ^d,e^	51.6 ^e^	0.027
Fat (g/kg)	3.4 ^d^	1.9 ^a,b^	2.0 ^b,c^	2.1 ^c^	1.8 ^a^	1.7 ^a^	1.9 ^a,b,c^	0.016
Total protein (g/kg)	9.6 ^e^	4.6 ^a^	4.9 ^a^	5.9 ^b^	6.8 ^c^	7.1 ^d^	5.6 ^b^	0.024
Lactose (g/kg)	37.8 ^a^	40.6 ^c^	40.5 ^b,c^	40.5 ^b,c^	40.2 ^b^	40.2 ^b^	41.8 ^d^	0.031
Non-fat solids/Total protein	5.3	10.6	10.1	8.7	7.5	7.2	9.2	
Fat/Total protein	0.4	0.4	0.4	0.4	0.3	0.2	0.3	
Ash (g/kg)	3.4 ^a^	3.9 ^b^	3.9 ^b^	3.9 ^b^	3.9 ^b^	3.9 ^b^	4.2 ^c^	0.006
pH	5.4 ^b^	5.3 ^a^	5.5 ^c^	5.6 ^c^	5.5 ^c^	5.6 ^c^	5.6 ^c^	0.002
Titratable acidity (% lactic acid)	0.325 ^e^	0.276 ^c,d^	0.272 ^b,c^	0.261 ^a^	0.279 ^d^	0.274 ^c,d^	0.267 ^b^	0.000

^a–e^ Means within a row with different superscripts differ (*p* < 0.05). SEM: standard error of the mean (*n* = 6). Sample coding as in Table 1.

**Table 6:** Cheese yield calculated for pasta filata cheeses from reconstituted sheep’s milk and mixtures of powder/reconstituted sheep’s milk and cow’s milk in different configurations.

Pasta Filata Cheese Yield									
Equation	Abbr.	Unit	SR	CSP6/4	CSP7/3	CSP8/2	CSR6/4	CSR7/3	CSR8/2
1	*X_SP_*	(%)	16.54	20.70	17.83	14.59	13.06	12.65	11.69
2	*X_MD_*		16.20	19.91	17.08	13.53	12.59	12.16	10.81
3	*X_H_*		18.06	21.85	19.85	18.12	19.42	18.77	18.55
4	*X_R_*		17.38	23.60	20.52	17.96	17.85	17.20	16.91
5	*X_J_*		20.05	25.75	22.18	18.11	19.60	18.64	16.99
6	*X_P_*		20.94	26.90	23.19	19.00	21.05	19.97	18.21
7	*MY*	(kg/kg)	5.11	5.67	5.58	5.54	4.33	4.64	4.81

Sample coding as in Table 1.

**Table 7:** Sensory acceptability of pasta filata cheeses from reconstituted sheep’s milk and mixtures of powder/reconstituted sheep’s milk and cow’s milk in different configurations.

Pasta Filata Cheese								
		SR	CSP6/4	CSP7/3	CSP8/2	CSR6/4	CSR7/3	CSR8/2
9	Like extremely (%)	8.82	0	0	0	0	9.80	1.96
8	Like very much (%)	23.53	0	0	0	0.98	58.82	11.76
7	Like moderately (%)	31.37	0	0	17.65	21.57	26.47	50.00
6	Like slightly (%)	7.84	0	0.98	26.47	45.10	3.92	35.29
5	Neither like nor dislike (%)	2.94	1.96	14.71	13.73	25.49	0.00	0.98
4	Dislike slightly (%)	9.80	7.84	11.76	19.61	3.92	0.98	0
3	Dislike moderately (%)	3.92	50.98	43.14	6.86	0.98	0	0
2	Dislike very much (%)	1.96	15.69	13.73	4.90	0.98	0	0
1	Dislike extremely (%)	9.80	23.53	15.69	10.78	0.98	0	0
Skewness		1.40	1.88	1.68	0.32	1.46	2.15	1.63
*p*-value		0.032	0.003	0.011	0.737	0.004	0.000	0.001
SD		10.11	17.49	14.15	9.27	16.36	20.28	19.01
CV		89.22	154.35	124.86	81.83	144.31	178.94	167.71
Dislike responses (%)		25.49	98.04	84.31	22.55	6.86	0.98	0

SD: standard deviation; CV: coefficient of variation; sample coding as in Table 1.

**Table 8:** Consumer penalty analysis of the just-about-right (JAR) diagnostic attributes of pasta filata cheeses from reconstituted sheep’s milk and mixtures of powder/reconstituted sheep’s milk and cow’s milk in different configurations.

Pasta Filata Cheese									
			SR	CSP6/4	CSP7/3	CSP8/2	CSR6/4	CSR7/3	CSR8/2
Appearance	Whey leachate	Not enough	–	–	–	–	–	–	–
		Too much	20.62	–	–	11.34	25.77	28.87	36.08
Consistency	Springiness	Not enough	19.59	11.34	10.31	–	–	–	–
		Too much	–	–	–	–	11.34	17.53	22.68
Aroma	Creamy	Not enough	–	–	-	18.56	18.56	30.93	42.27
		Too much	16.49	27.84	23.71	20.62	23.71	15.46	–
Flavor	Salty	Not enough	–	–	–	–	–	–	12.37
		Too much	18.56	14.43	13.40	–	17.53	11.34	11.34

(–): indicates that less than 10% of consumers chose that JAR category; sample coding as in Table 1.

## Data Availability

The authors confirm that the data supporting the findings of this study are available within the article.
